# Novel high molecular weight polymerized hemoglobin in a non-obese model of cardiovascular and metabolic dysfunction^☆^

**DOI:** 10.1016/j.biopha.2024.116789

**Published:** 2024-05-29

**Authors:** Cynthia R. Muller, Alexander T. Williams, Allyn M. Eaker, Cynthia Walser, Fernando Dos Santos, Clayton T. Cuddington, Savannah R. Wolfe, Andre F. Palmer, Pedro Cabrales

**Affiliations:** a Department of Bioengineering, University of California, San Diego, CA, USA; b Department of Anesthesiology & Critical Care, University of California, San Diego, CA, USA; c William G. Lowrie Department of Chemical and Biomolecular Engineering, The Ohio State University, Columbus, OH, USA

**Keywords:** High-fat high sucrose diet, Metabolic disorders, Hemoglobin-based oxygen carriers, Red blood cell substitute, Oxygen therapeutic, Polymerized hemoglobin

## Abstract

The widespread adoption of high-calorie, high-fat, high-sucrose diets (HFHSD) has become a global health concern, particularly due to their association with cardiovascular diseases and metabolic disorders. These comorbidities increase susceptibility to severe outcomes from viral infections and trauma, with trauma-related incidents significantly contributing to global mortality rates. This context underscores the critical need for a reliable blood supply. Recent research has focused on high molecular weight (MW) polymerized human hemoglobin (**PolyhHb**) as a promising alternative to red blood cells (**RBCs**), showing encouraging outcomes in previous studies. Given the overlap of metabolic disorders and trauma-related health issues, it is crucial to assess the potential toxicity of PolyhHb transfusions, particularly in models that represent these vulnerable populations. This study evaluated the effects of PolyhHb exchange transfusion in guinea pigs that had developed metabolic disorders due to a 12-week HFHSD regimen. The guinea pigs, underwent a 20 % blood volume exchange transfusion with either PolyhHb or the lower molecular weight polymerized bovine hemoglobin, Oxyglobin. Results revealed that both PolyhHb and Oxyglobin transfusions led to liver damage, with a more pronounced effect observed in HFHSD-fed animals. Additionally, markers of cardiac dysfunction indicated signs of cardiac injury in both the HFHSD and normal diet groups following the Oxyglobin transfusion. This study highlights how pre-existing metabolic disorders can exacerbate the potential side effects of hemoglobin-based oxygen carriers (HBOCs). Importantly, the newer generation of high MW PolyhHb showed lower cardiac toxicity compared to the earlier generation low MW PolyhHb, known as Oxyglobin, even in models with pre-existing endothelial and metabolic challenges.

## 1. Introduction

The consumption of high-calorie and high-fat, high-sucrose diets (HFHSD) has increased globally in recent decades, posing a significant worldwide health concern [1]. These diets have been linked to cardiovascular disease (CVD) and metabolic disorders such as dyslipidemia, diabetes, and non-alcoholic fatty liver disease (NAFLD) [1]. Individuals with these comorbidities are more vulnerable to adverse outcomes when attempting to recover from infections, such as COVID-19 [2], and experience worse outcomes after resuscitation from trauma [3]. While CVD and metabolic disorders are typically associated with obesity, other studies have highlighted the importance of diet in developing comorbidities independent of obesity [4,5]. Poor diet is associated with NAFLD, and there is a correlation between NAFLD and an increased risk for CVD [4,5]. Our group has recently developed and characterized a high-fat, high-sucrose diet guinea pig model, which presents cardiovascular and metabolic disorders independent of obesity [6].

Trauma accounts for a significant percentage of annual deaths worldwide, and a considerable number of patients require blood transfusions for trauma resuscitation [7]. Unfortunately, ensuring a consistent and secure blood supply remains a significant challenge. Red blood cells (RBCs) have a limited shelf life of 42 days, depending on the specific additive solution employed. This temporal constraint complicates the delicate task of maintaining an optimal equilibrium between blood storage and utilization, leading to issues such as over-donation and, conversely, blood shortages [8].

Numerous efforts have been undertaken to develop alternative acellular and encapsulated types of hemoglobin (Hb)-based oxygen carriers (HBOCs) [9]. Several iterations of HBOCs have been devised, including alpha-alpha cross-linked Hb (ααHb), polyethylene glycol-surface conjugated Hb (PEG-Hb), polymerized Hb (PolyHb), and encapsulated Hb [9,10]. Polymerization and encapsulation are the most frequent processes that increase intravascular retention and prolong circulating time while decreasing the toxicity and vasoactivity of acellular Hb. PolyHb can be synthesized in either the fully deoxygenated state (pO_2_ = 0 mm Hg), comprising 100 % T-quaternary state Hb, or the fully oxygenated state (pO_2_ = 760 mm Hg), comprising 100 % R-quaternary state Hb [11]. Furthermore, non-Hb-containing modalities have been explored, such as perfluorocarbons (PFCs), a class of compounds with a high solubility for both oxygen and carbon dioxide. The revelation that PFCs could dissolve a significant amount of oxygen, albeit at high oxygen partial pressures, suggested their potential use as blood substitutes [10].

Although there is a pressing need to develop alternatives to RBCs, HBOCs and previous generations of HBOCs demonstrated efficacy in experimental animal models; severe adverse safety signals in phase II and phase III clinical trials hindered further commercial development [12,13]. Recently, high molecular weight (MW) polymerized human Hb (PolyhHb) has been evaluated with positive results in various experimental animal models, demonstrating its ability to restore oxygenation with reduced or minimal vasoconstriction or hypertension [14–16]. Our group has shown that increasing the molecular size of PolyhHb decreases hypertension and minimizes Hb-induced toxicity in healthy guinea pigs [16]. Guinea pigs are a more clinically relevant model than other rodents as they, along with humans and other primates, cannot synthesize ascorbic acid in their liver [17,18].

Recently, studies have shown promising results regarding the ability of high MW PolyhHb to restore hemodynamics similarly to blood in a model of traumatic brain injury (TBI) followed by severe hemorrhagic shock (HS) in rats [14]. However, that study was conducted in healthy animal models without pre-existing conditions. Therefore, it is essential to consider how PolyhHb may affect populations with existing health conditions, as a significant percentage of the population has CVD or metabolic syndrome. Estimates indicate that the global prevalence of metabolic syndrome varied from 12.5–31.4 %, according to the World Health Organization (WHO) definition considered in the report. The prevalence of metabolic syndrome was significantly higher in the Eastern Mediterranean Region and Americas and increased with a country’s income level. The global prevalence was 45.1 % for central obesity, 42.6 % for systolic blood pressure (BP) ≥ 130 mmHg, and diastolic BP ≥ 85 mmHg [19].

Given these high incidence rates, it is critical to study the potential toxicity of blood transfusions and blood alternatives such as PolyhHb in models reflecting vulnerable populations. Therefore, the present study aimed to evaluate the effects of high MW PolyhHb exchange transfusion in a metabolic disorder model induced by a high-fat, high-sucrose diet in guinea pigs. In this model, we compared the side effects from PolyhHb to a previous generation HBOC (Oxyglobin) after exchange transfusion to test the hypothesis that HFHSD guinea pigs may be more susceptible. Evaluating PolyhHb toxicity in cardiovascular and metabolic disease models will provide important safety data to guide the translation and clinical use of this promising blood substitute.

## 2. Methods

### 2.1. Animal preparation

The researchers followed the National Institutes of Health Guide for the Care and Use of Laboratory Animals for Animal Handling and Care, and the local Animal Care Committee approved the experimental protocol. Guinea pigs (GPs) were obtained from Charles River USA weighing between 200 and 220 g. GPs were fed an HFHSD (Envigo TD.110484; 35 % sucrose, 15 % cocoa butter, 0.25 % cholesterol) for 12 weeks [6]. Hemodynamic and hematological parameters were comparable with previous results with this diet [6]. The animals were placed on a heating pad to maintain core body temperature at 37 °C for the duration of the experiment under anesthesia. In this study, we utilized a total of 72 guinea pigs, evenly distributed across 6 groups, with 12 animals per group. The variance in the number of animals among groups in the final results is attributable to the intricacies and multiple steps required to conduct all experiments.

### 2.2. High MW polymerized human hemoglobin

PolyhHb was synthesized in the low O_2_ affinity tense (T) state at a 30:1 molar ratio of glutaraldehyde to hemoglobin (Hb). The PolyhHb was then filtered through a 0.2 μm hollow fiber filter, and then subjected to 8–9 cycles of diafiltration on a 500 kDa hollow fiber filter. This resulted in a PolyhHb solution containing only polymerized Hb molecules bracketed between 500 kDa – 0.2 μm. PolyhHb synthesis and characterization followed techniques described in the literature [20–22].

### 2.3. Oxyglobin

Oxyglobin^®^ (HBOC-301, hemoglobin glutamer-200, Biopure Corporation) is an HBOC that increases plasma and total Hb concentrations and thus increases arterial blood O_2_ content. The plasma half-life is 30–40 hours.

### 2.4. Exchange transfusion

GPs were anesthetized with isoflurane (Drägerwerk AG, Lübeck, Germany) slowly, by increasing the isoflurane 0.4 % every 3 minutes until a surgical depth of anesthesia was achieved, typically 3 %. This ensured that the animals did not stop breathing due to airway irritation by isoflurane, and prevented variations in heart rate (HR). Animals were instrumented with catheters in the right carotid artery and left jugular vein, and catheters were exteriorized dorsally. Anesthetized GPs were randomized before the animals were subjected to a 20 % exchange transfusion (ET) (based on the estimated blood volume of 7.5 % of body weight) with the study solutions infused at a rate of 750 μL/min. The Sham group was subjected to the same surgical procedure but was not exchanged-transfused. Normal diet and HFHSD diet animals were randomly assigned to one of the three experimental groups. Sham, PolyhHb (10 g/dL), or Oxyglobin (12 g/dL). After ET, all animals were allowed to recover, and additional hemodynamics and toxicological measurements were taken 24 hours after ET.

### 2.5. Hematological parameters

Hematocrit (Hct) was measured from centrifuged arterial blood samples taken in heparinized capillary tubes. Arterial blood was collected in heparinized glass capillaries (50 μL) and immediately analyzed for pO_2_, pCO_2_, pH, and Hb content (ABL90; Radiometer America, Brea, CA).

### 2.6. Blood pressure, heart rate (HR), heart rate variability (HRV), blood pressure variability (BPV) and spectral analysis

Twenty-four hours after ET, the arterial cannula was connected to a pressure transducer (MP150, Biopac, Santa Barbara, CA) to record continuous blood pressure (BP) and heart rate (HR) signals for 10 min. Heart rate variability (HRV) was calculated in time and frequency domains using the Cardioseries^®^ v2.4 software (Ribeirão Preto, SP, Brazil). The BP and HR were recorded in awake GPs for 10 minutes for analysis of pulse interval (PI) and systolic blood pressure (SBP) in the time domain, obtaining the total variance of PI (VAR PI) and the variance of SBP (VAR SBP). For the frequency domain, the interpolated waves of these same baseline periods were divided into segments of 512 beats, with an overlap of 50 %, and were processed by Fast Fourier Transform. One spectrum was obtained for each segment and the oscillatory components were quantified in two different frequencies: low frequency (LF) from 0.10 to 0.75 Hz and high frequency (HF) from 0.75 to 3.00 Hz. The results are represented by absolute values (ms^2^ and mmHg^2^), the percentage of total spectrum (%), and normalized units (nu) (percentage of LF and HF bands only). The very low oscillations (<0.20 Hz) were considered non-stationary [23].

### 2.7. Vasoreactivity measurements

Twenty-four hours after ET and after completing BP and HR variability analysis, changes in endothelial function and nitric oxide (NO) sensitivity were evaluated. Endothelial function and NO sensitivity were studied using the changes in mean arterial pressure (MAP) in response to infusion of acetylcholine (2 and 4 μg, with a 10-minute interval between doses, Sigma Aldrich) and via administration of the nitric oxide synthase (NOS) inhibitor N(ω)-nitro-L-arginine methyl ester (L-NAME, 12 mg/kg, Sigma Aldrich).

### 2.8. Harvesting tissues

Two hours after vasoreactivity measurements, the animals were anesthetized, and blood was collected and then centrifuged to separate RBCs and plasma. Lastly, animals were euthanized with Fatal Plus^®^ (sodium pentobarbital, 300 mg/kg), and quickly urine was collected, and vital tissues (kidney, liver, spleen, heart, and lung) were harvested. Markers of inflammation, organ function, and organ injury were evaluated. These analyses were performed by the UC San Diego Histology Core via ELISA and flow cytometric analysis of tissue homogenates and plasma. The kits and methods used for these analyses are described in Supplementary Table 1.

### 2.9. Statistical analysis

All values are expressed as mean ± SE. Data were analyzed using a Two-Way Analysis of Variance (ANOVA). When appropriate, post hoc analyses were performed with the Tukey multiple comparisons test. All statistics were calculated using GraphPad Prism 9 (GraphPad Software, Inc., San Diego, CA). Changes were considered significant if p < 0.05.

## 3. Results

### 3.1. Solution biophysical properties

High MW PolyhHb and Oxyglobin biophysical properties are presented in Table 1. High MW PolyhHb at 10 g/dL has a higher solution viscosity than Oxyglobin at 12 g/dL. Oxyglobin is an HBOC that increases plasma and total hemoglobin concentrations and thus increases arterial blood oxygen content. The plasma half-life is 30–40 hours. This formulation processed 6.4 % of species with MW > 500 kDa, 27.1 % of species ~65 kDa (tetrameric Hb), 3.8 % of species with MW < 32 kDa, and 30.9 % of mixed tetramer + dimer [24]. It contains less than the detectable level of 3.5 μg/mL free glutaraldehyde and 0.05 EU/mL endotoxin.

All animals survived the entire experimental protocol. BP and HR were not studied during anesthesia as guinea pigs are very sensitive to anesthesia. All animals recovered from anesthesia within a similar time frame. Hypothermia was prevented during anesthesia using a circulating warm-water blanket.

#### Hematological Parameters. GPs fed an HFHSD.

Hematological parameters such as blood gases, electrolytes, total Hb (tHb), and lactate are presented in Table 2.

There was no statistical difference in tHb, pH, or pO_2_ between the HFHSD-Sham and ND-Sham groups. pCO_2_ was slightly lower for the HFHSD-Sham group compared to the ND-Sham group. Also, sodium and chloride concentrations in the blood were statistically lower in the HFHSD-Sham group compared to the ND-Sham group, but no further changes were observed in the potassium, calcium, and lactate in between these groups.

Exchange transfusion with PolyhHb and Oxyglobin decreased tHb in the groups fed with a ND compared to the ND-Sham group, however, exchange transfusion did not cause any further changes in electrolytes or blood gases in the exchange transfused ND groups.

Similarly, exchange transfusion with both PolyhHb and Oxyglobin decreased tHb in the HFHSD groups compared to the HFHSD-Sham group. There was an increase in chloride and lactate blood concentrations in the HFHSD-Oxyglobin group compared to the HFHSD-Sham group, furthermore, lactate was higher in the HFHSD-Oxyglobin group compared to the HFHSD-PolyhHb group. No further statistically significant differences were observed for electrolytes and blood gases between the exchange transfused HFHSD groups.

The HFHSD-PolyhHb group presented lower pCO_2_, sodium, and chloride compared to the ND-PolyhHb group, and the HFHSD-Oxyglobin group presented lower levels of sodium and higher levels of lactate compared to the ND-Oxyglobin group.

#### Systemic Hemodynamic Parameters.

**Fig. 1.**
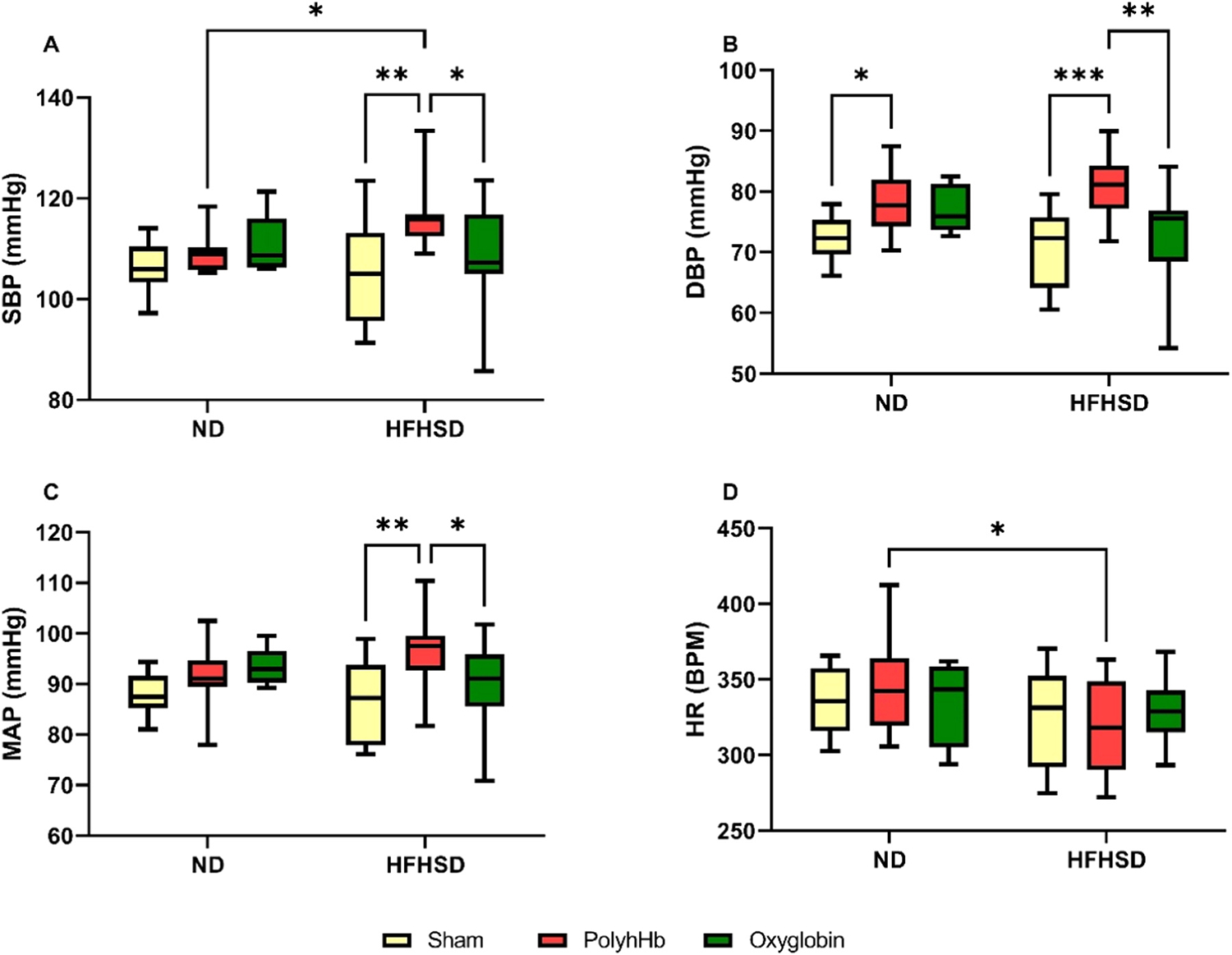
Blood pressure measurements of normal diet (ND) or high-fat high-sucrose diet (HFHSD) animals for the Sham group, and 20 % exchange transfusion with the PolyhHb or Oxyglobin groups. A- Systolic blood pressure (SBP); B- Diastolic blood pressure (DBP); C- Mean arterial pressure (MAP); D- Heart rate (HR). ND-Sham (n = 7); ND-PolyhHb (n=7); ND-Oxyglobin (n=9); HFHSD-Sham (n = 5); HFHSD-PolyhHb (n=8); HFHSD-Oxyglobin (n=12). *p<0.05; **p<0.01; ***p<0.001.

Fig. 1 shows the results for BP and HR. There was no statistically significant difference in SBP, DBP, MAP, and HR between the HFHSD-Sham group compared to the ND-Sham group.

Exchange transfusion with PolyhHb increased DBP in the ND-PolyhHb group compared to the ND-Sham group, but no further differences were observed in the ND animals when submitted to an exchange transfusion with PolyhHb or Oxyglobin.

Exchange transfusion with PolyhHb increased SBP, DBP, and MAP in the HFHSD-PolyhHb group compared to the HFHSD-Sham group. Exchange transfusion with Oxyglobin did not cause any significant changes in BP parameters in the HFHSD-Oxyglobin group compared to the HFHSD-Sham group. Also, HFHSD animals exchange transfused with Oxyglobin presented statistically lower SBP, DBP, and MAP compared to the HFHSD-PolyhHb group.

The HFHSD-PolyhHb group presented higher SBP and lower HR compared to the ND-PolyhHb group. However, no statistically significant difference was observed between ND and HFHSD groups exchanged with Oxyglobin.

#### Vasoreactivity Measurements.

**Fig. 2.**
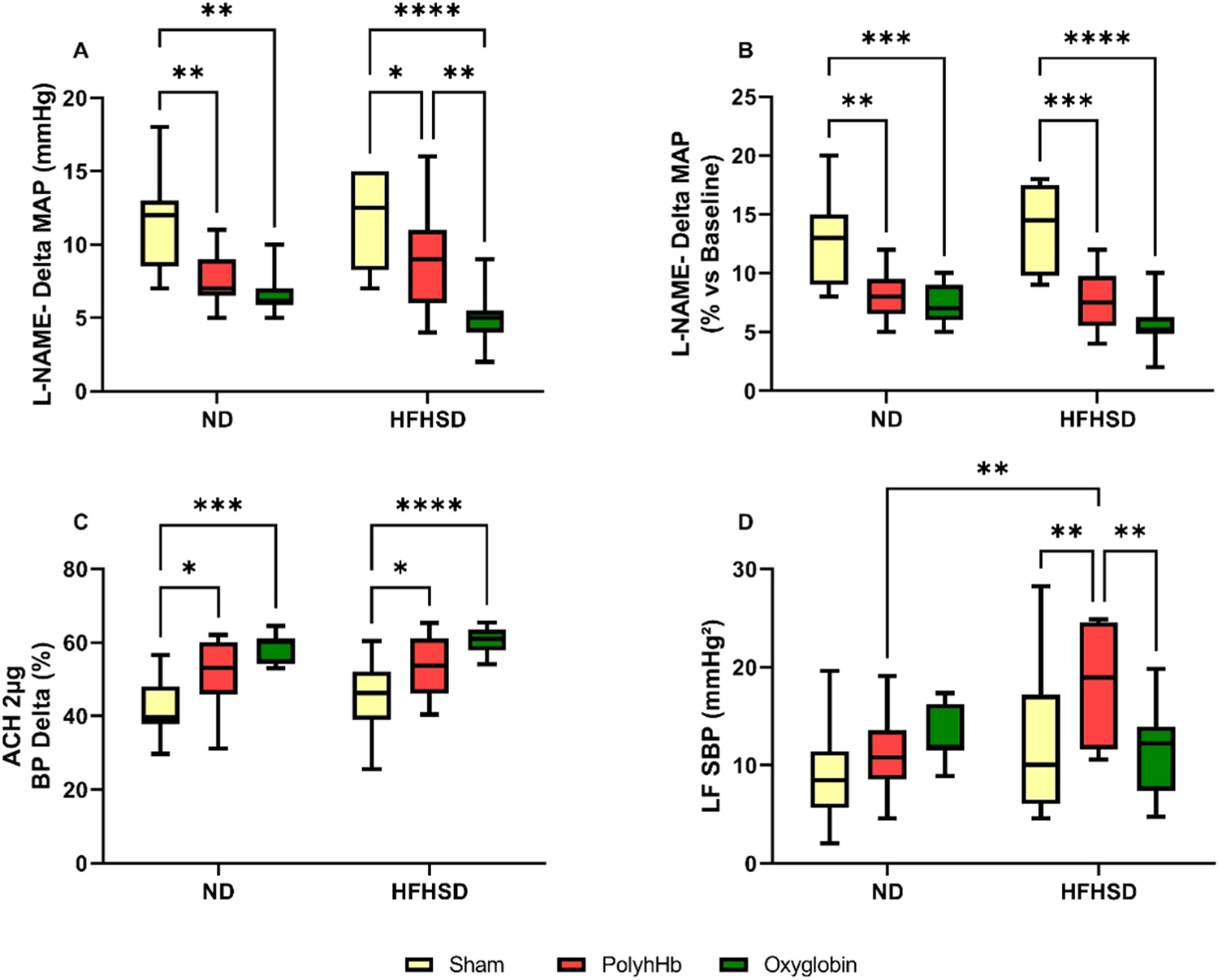
Vascular measurements of normal diet (ND) or high-fat high-sucrose diet (HFHSD) animals for the Sham group, and the 20 % exchange transfusion with the PolyhHb or Oxyglobin groups. A- MAP response to L-NAME (mmHg); B- MAP response to L-NAME (mmHg %); C- MAP response to ACH (%); and D- Low frequency band of systolic blood pressure (LF SBP) (mmHg^2^). ND-Sham (n = 7); ND-PolyhHb (n=7); ND-Oxyglobin (n=7); HFHSD-Sham (n = 5); HFHSD-PolyhHb (n=8); HFHSD-Oxyglobin (n=9). *p<0.05; **p<0.01; ***p<0.001; ****p<0.0001.

To evaluate the responses to NO scavenging, animals were challenged with L-NAME. And the results are presented in Fig. 2 panels A-B.

There were no differences in the response to L-NAME in the HFHSD-Sham group compared to the ND-Sham group.

Exchange transfusion with both PolyhHb and Oxyglobin decreased the L-NAME response in the ND-PolyhHb and ND-Oxyglobin groups when compared to the ND-Sham group, without differences between ND-PolyhHb and ND-Oxyglobin, indicating a certain degree of NO scavenging.

In the same manner, exchange transfusion with both PolyhHb and Oxyglobin decreased the BP response to L-NAME in the HFHSD-PolyhHb and HFHSD-Oxyglobin groups when compared to the HFHSD-Sham group. However, the HFHSD-Oxyglobin group showed a slightly higher decreased response when compared to the HFHSD-PolyhHb group.

No differences were observed in the L-NAME response between the HFHSD-PolyhHb and ND-PolyhHb groups or ND-Oxyglobin and HFHSD-Oxyglobin groups.

In an attempt to determine endothelial dysfunction, these animals were also submitted to a challenge with acetylcholine (ACH), a vasodilator (Fig. 2 panel C). The response to ACH was not different between the HFHSD-Sham and ND-Sham groups.

Exchange transfusion with both solutions in the ND group increases the % of BP decay equally when compared to the ND-Sham group. In the same manner, the exchanged transfused HFHSD groups presented similar BP decay after ACH injection, indicating a higher response to ACH in both HFHSD exchanged groups when compared to the HFHSD-Sham group.

No difference was observed between the ND-PolyhHb and ND-Oxyglobin groups or the HFHSD-PolyhHb and HFHSD-Oxyglobin groups.

#### BP and HRV analysis.

*The complete* BP and HR variability and autonomic nervous system analysis are shown in Supplemental Table 2, while the sympathetic modulation of the blood pressure is presented in Fig. 2 **Panel D**.

The sympathetic modulation of the blood pressure was increased only in the HFHSD-PolyhHb group when compared to the other HFHSD groups, and to ND-PolyhHb group (Fig. 2 **Panel D**).

BP variability was increased in the HFHSD-Sham group compared to the ND-Sham group.

Exchange transfusion with PolyhHb and Oxyglobin equally caused a cardiac autonomic imbalance observed by an increased sympathetic-vagal balance (LF/HF). This was caused by both an LF (sympathetic modulation) increase, and an HF (parasympathetic modulation) decrease in the ND-exchanged transfused groups when compared to the ND-Sham group. No differences were observed between ND-PolyhHb and ND Oxyglobin.

Exchange transfusion with PolyhHb or Oxyglobin did not cause any remarkable change in BP and HR variability or autonomic nervous system in the HFHSD groups when compared to the HFHSD-Sham group. However, exchange transfusion with Oxyglobin decreased the LF/HF balance resulting from a decreased LF and increased HF compared with the HFHSD-PolyhHb group.

More importantly, exchange transfusion with Oxyglobin in the HFHSD group caused an increase in BP and HR variability when compared to ND-Oxyglobin, however, the sympathetic-vagal balance was decreased in the HFHSD-Oxyglobin group when compared to the ND-Oxyglobin group.

#### Organ Injury and Systemic Inflammation.

##### Liver Injury:

**Fig. 3.**
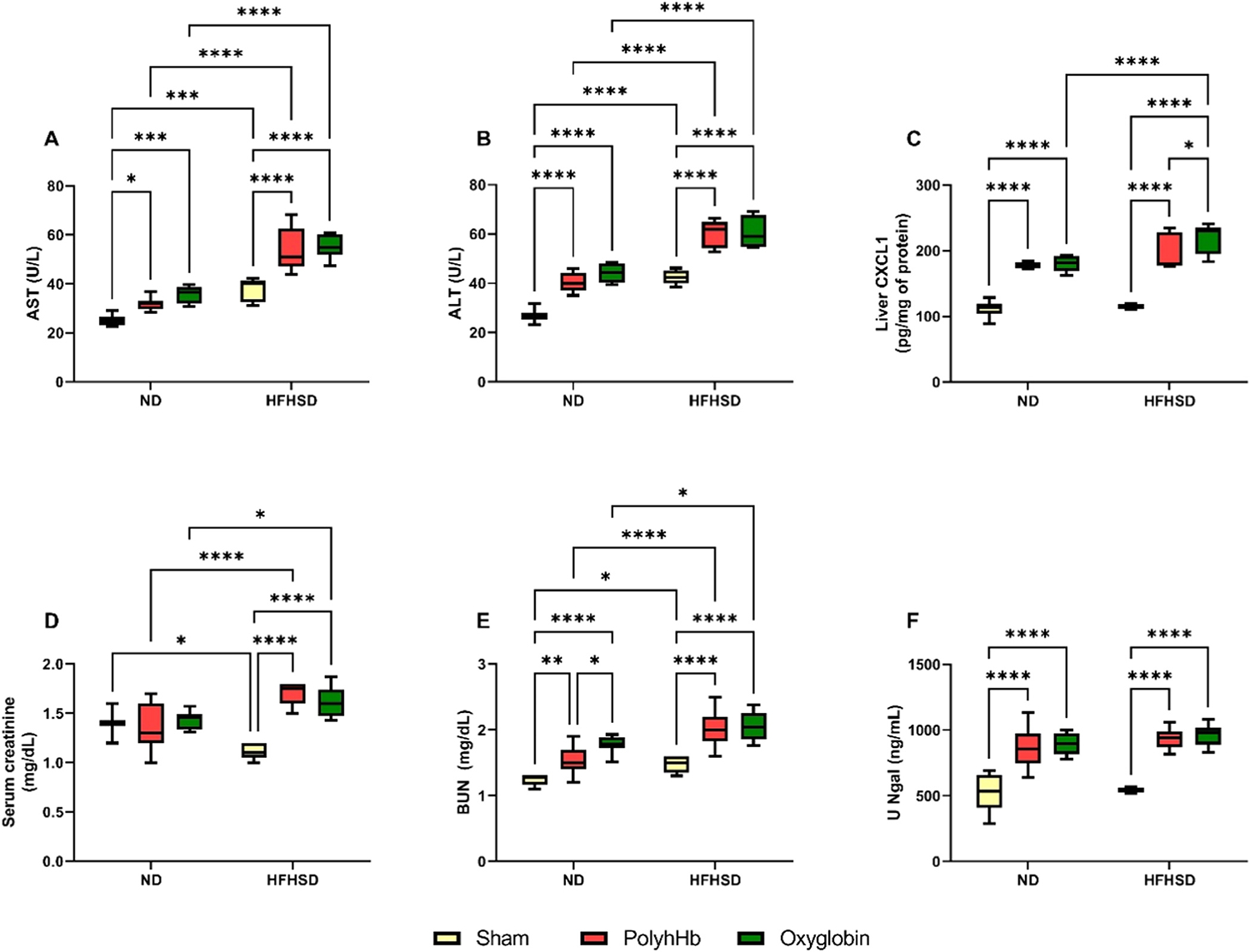
Liver and kidney injury after 12 weeks of normal diet (ND) or high-fat high-sucrose diet (HFHSD) for the Sham group or 20 % exchange transfusion with PolyhHb or Oxyglobin groups. A- Aspartate aminotransferase (AST); B- Alanine aminotransferase (ALT); C- Liver chemokine ligand- 1 (CXCL-1); D- Serum creatinine; E- Blood urea nitrogen (BUN); F- Urine neutrophil gelatinase-associated lipocalin (U-NGAL). ND-Sham (n = 7); ND-PolyhHb (n=7); ND-Oxyglobin (n=8); HFHSD-Sham (n = 5); HFHSD-PolyhHb (n=8); HFHSD-Oxyglobin (n=8). *p<0.05; **p<0.01; ***p<0.001; ****p<0.0001.

Liver enzymes are represented in Fig. 3 Panel A-C, liver aspartate aminotransferase (AST) and alanine transaminase (ALT) were increased in the HFHSD-Sham group compared to the ND-Sham group, without any difference in the inflammatory marker liver chemokine ligand- 1 (CXCL-1) between these groups.

Exchange transfusion with both PolyhHb and Oxyglobin in the ND animals increased AST, ALT, and CXCL-1. However, no differences were observed between ND-PolyhHb and ND-Oxyglobin, for these 3 liver damage parameters.

In the same manner, exchange transfusion with both solutions increased AST, ALT, and CXCL-1 compared to the HFHSD-Sham group. It is important to note that Oxyglobin increased CXCL-1 even more in the HFHSD-Oxyglobin group compared to the HFHSD-PolyhHb group.

Moreover, the HFHSD-PolyhHb group presented higher levels of AST and ALT compared to the ND-PolyhHb group, and the HFHSD-Oxyglobin group increased AST, ALT, and CXCL-1 compared to the ND-Oxyglobin group.

#### Kidney Injury.

Markers of kidney injury are shown in Fig. 3 Panels DF. The HFHSD-Sham group presented lower serum creatinine and increased levels of BUN compared to the ND-Sham group. However, no change was observed in the levels of U Ngal between these 2 groups.

Exchange transfusion with PolyhHb or Oxyglobin did not change serum creatinine in the ND-exchanged transfused animals. On the other hand, both solutions increased BUN and U Ngal when compared in the ND groups to the ND-Sham group. Finally, the ND-Oxyglobin group presented higher BUN compared to the ND-PolyhHb group.

Exchange transfusion with both PolyhHb and Oxyglobin increased serum creatinine, BUN, and U Ngal in the animals fed an HFHSD when compared to the Sham group, however, no differences were observed for these 3 markers between the HFHSD-PolyhHb and HFHSD-Oxyglobin groups, suggesting that both solutions are equally causing some kidney damage.

HFHSD-PolyhHb had higher levels of serum creatinine and BUN when compared to the ND-PolyhHb group, without changing U Ngal. Similarly, we observed increased levels of serum creatinine and BUN in the HFHSD-Oxyglobin group when compared to the ND-Oxyglobin group.

#### Systemic Inflammation and Catecholamines.

Table 3 shows markers of systemic and splenic inflammation, as well as catecholamines. No changes in systemic inflammatory markers were observed in the HFHSD-Sham group when compared to the ND-Sham group. Epinephrine was increased in the HFHSD-Sham group compared with the ND-Sham group while norepinephrine remained the same.

Exchange transfusion with both PolyhHb and Oxyglobin increased IL-6, IL-10, and systemic CXCL1 in ND animals compared to ND-Sham animals. Norepinephrine was increased in the ND-Oxyglobin group compared to the ND-Sham group, but no changes were observed for the ND-PolyhHb group. On the other hand, epinephrine increased for both the ND-PolyhHb and ND-Oxyglobin groups compared to the ND-Sham group. Moreover, the ND-Oxyglobin group presented even higher plasma CXCL-1 levels compared to the ND-PolyhHb group.

Exchange-transfusion with PolyhHb and Oxyglobin also increased inflammatory markers and catecholamines in the animals fed an HFHSD, observed by increased IL-6, IL-10, plasma and splenic CXCL1, epinephrine, and norepinephrine. No differences were observed for these markers between the HFHSD-PolyhHb and HFHSD-Oxyglobin groups.

It is important to note that all the inflammatory markers and catecholamines were higher for the HFHSD-PolyhHb group compared to the ND-PolyhHb group, as well as for the HFHSD-Oxyglobin group compared to the ND-Oxyglobin group, suggesting that animals fed an HFHSD are more susceptible to exchange transfusion inflammatory side effects than ND animals.

#### Iron Metabolism.

Ferritin and bilirubin were measured to evaluate iron and Hb metabolism after exchange transfusion and the results are shown in Table 3. It was observed that the total bilirubin in the HFHSD-Sham group increased compared to the ND-Sham group, but no changes in serum, liver, or spleen ferritin were observed between these groups.

Exchange transfusion with PolyhHb and Oxyglobin increased equally serum, liver, and spleen ferritin in the ND-exchanged transfused animals compared to the ND-Sham group, however, only ND-Oxyglobin increased total bilirubin compared to the ND-Sham group.

Exchange transfusion with either PolyhHb or Oxyglobin increased serum, liver, and spleen ferritin, as well as total bilirubin in the HFHSD exchanged groups when compared to the HFHSD-Sham group. Furthermore, no differences were observed between HFHSD-PolyhHb and HFHSD-Oxyglobin groups.

It is important to highlight that ferritin was lower for the HFHSD-PolyhHb group compared to the ND-PolyhHb group, and also lower for the HFHSD-Oxyglobin groups when compared to the ND-Oxyglobin group, indicating that the HFHSD is preventing these animals from increasing ferritin after exchange transfusion at the same levels as a healthy (ND diet) animal.

#### Cardiac Damage.

**Fig. 4.**
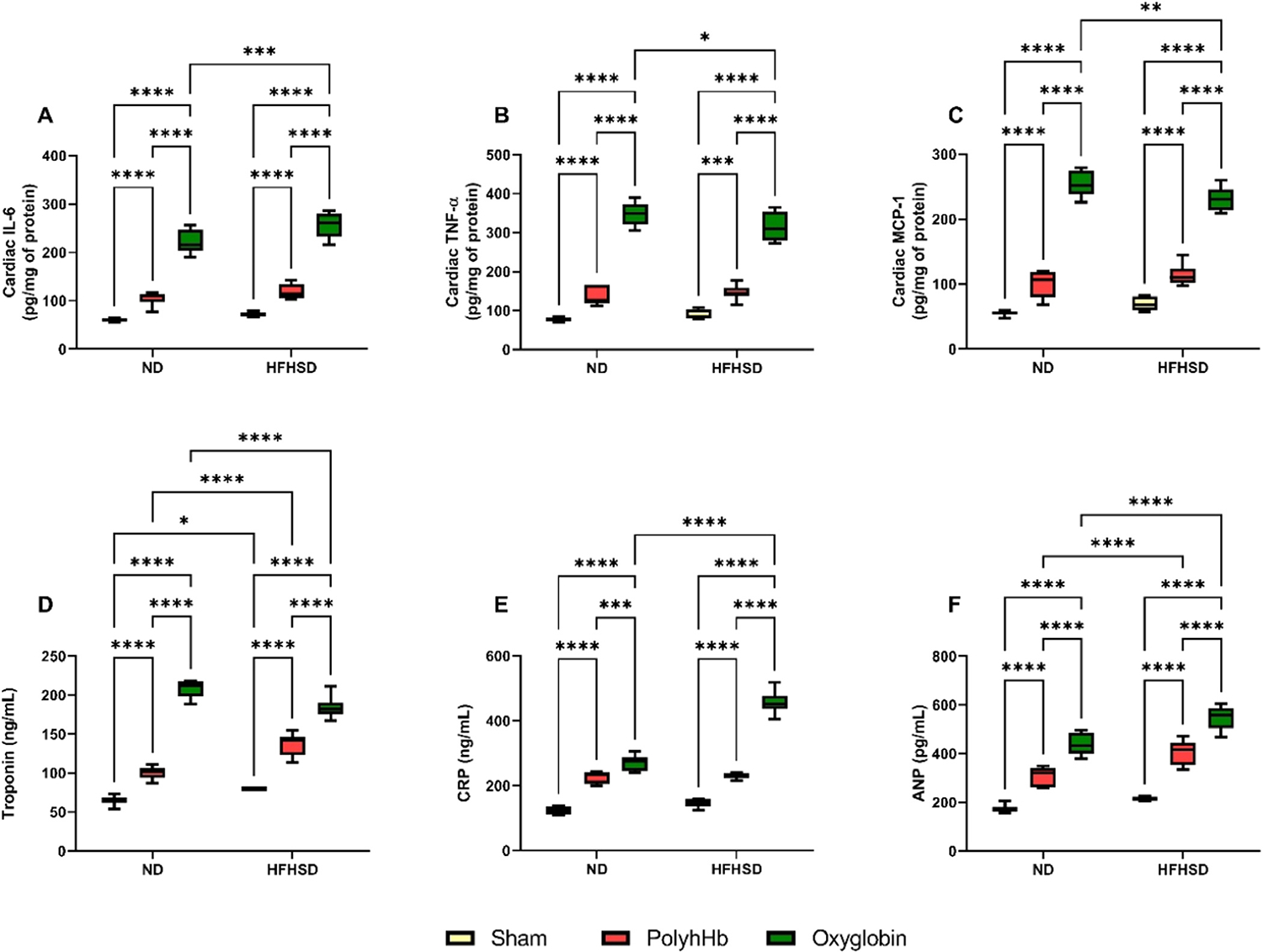
Cardiac inflammation and injury after 12 weeks of normal diet (ND), and high-fat high-sucrose diet (HFHSD) for the Sham group or 20 % exchange transfusion with the PolyhHb or Oxyglobin groups. A- Cardiac interleukin- 6 (IL-6); B- Cardiac tumor necrosis factor alpha (TNF-α); C- Cardiac monocyte chemoattractant protein-1 (MCP-1); D- Cardiac troponin; E- C-reactive protein (CRP); and F- Atrial natriuretic peptide (ANP). ND-Sham (n = 7); ND-PolyhHb (n=7); ND-Oxyglobin (n=8); HFHSD-Sham (n = 5); HFHSD-PolyhHb (n=8); HFHSD-Oxyglobin (n=8). *p<0.05; **p<0.01; ***p<0.001; ****p<0.0001.

To indirectly evaluate cardiac dysfunction, several markers were measured, the results are represented in Fig. 4 Panels A-F, and it was observed that there were higher levels of troponin in the HFHSD-Sham group compared to the ND-Sham group, but no further changes were found for these markers between these groups.

Exchange transfusion with either PolyhHb or Oxyglobin increased all the cardiac damage markers compared to the ND-Sham group. Moreover, exchange transfusion increased, even more, the cardiac damage markers in the ND-Oxyglobin group compared to the ND-PolyhHb group, except for CRP, which remained the same between these two groups.

Similarly, animals fed an HFHSD and exchanged transfused with PolyhHb or Oxyglobin increased all the cardiac damage markers compared to the HFHSD-Sham group. And importantly all these markers were even higher for the HFHSD-Oxyglobin group compared to the HFHSD-PolyhHb group. Suggesting that exchange transfusion with Oxyglobin is causing higher cardiac toxicity in both ND and HFHSD animals.

It was observed that there was higher cardiac troponin and ANP when comparing the HFHSD-PolyhHb group to the ND-PolyhHb group with no further changes in the other parameters. Curiously, when comparing the HFHSD-Oxyglobin group to the ND-Oxyglobin group, there was increased IL-6, CRP, and ANP, but decreased IL-10, MCP-1, and troponin.

## 4. Discussion

In this study, we evaluated PolyhHb in a model with a pre-existing condition caused by an HFHSD. We previously showed guinea pigs fed an HFHSD for 12 weeks developed dyslipidemia, glucose intolerance, and cardiac damage [6], which we believe could predispose these animals to more severe side effects when exchange transfused with PolyhHb. Here, we demonstrated that guinea pigs with HFHSD-induced metabolic disorders, rather than healthy guinea pigs on a normal diet, were more susceptible to developing organ damage and autonomic dysfunction when exchanged transfused with PolyhHb. More importantly, we demonstrated that PolyhHb has lower cardiac toxicity than exchange transfusion with Oxyglobin (HBOC 301), a prior-generation polymerized Hb product, in animals with pre-existing conditions. As described previously, earlier generations of HBOCs similar to Hemopure failed clinical trials due to side effects such as vasoconstriction and cardiovascular risks [12,13]. Oxyglobin, tested in this study, is similar to Hemopure but has a lower MW.

This study was based on the hypothesis that increasing the MW of PolyhHb could decrease its toxicity. Infusion of higher molecular weight PolyhHb results in less free Hb extravasation through the endothelium, which mitigates toxicity, NO scavenging, and iron tissue accumulation. This reduces toxicity, as our previous publications show [15,25,26]. PolyhHb also performs similarly to fresh blood during hemorrhagic shock and causes less damage than stored blood [25]. However, our previous studies were in healthy (normal dieted) animals. The goal of this new study was to test if this new generation of PolyhHb would be safe in animals with pre-existing conditions. Evaluating a model with HFHSD-induced metabolic disorders is important because it represents a significant portion of the population [19]. Our previous work showed that GPs fed an HFHSD for 12 weeks developed dyslipidemia, glucose intolerance, and cardiac damage, which could potentially predispose them to more severe side effects when exchange transfused with PolyhHb [16]. As highlighted earlier, this study demonstrated the high MW PolyhHb reduced cardiac toxicity compared to Oxyglobin in both normal and HFHSD animals. Testing PolyhHb safety and efficacy in a model of diet-induced metabolic disorders is crucial in guiding the clinical translation of this promising RBC substitute. Evaluating the improved safety profile in animals with pre-existing conditions will provide essential data to support use in patient populations with common cardiovascular and metabolic diseases.

The decreased tHb observed after exchange transfusion with PolyhHb was an expected effect, independent of diet, as described in our previous publications. This decrease in tHb occurs because of PolyhHb clearance from the blood [15], and this decrease may be happening for the same reason in the Oxyglobin groups. Changes in electrolytes are an effect of the HFHSD itself rather than due to the PolyhHb or Oxyglobin. Similar electrolyte changes were observed in our initial study, characterizing the impact of the HFHSD on GPs without any exchange transfusion [6]. Although the electrolyte changes were statistically significant in the current study, the levels remained within normal physiological ranges and are unlikely to cause further physiological disturbances.

Plasma norepinephrine and epinephrine were markedly increased in HFHSD animals exchanged with both PolyhHb and Oxyglobin solutions. Stress hormones indicate significant activation of the sympathetic nervous system (SNS), which further demonstrates that the metabolic disorders induced by the HFHSD made this group more susceptible to autonomic dysfunction following exchange-transfusion. Spectral analysis of systolic blood pressure (SBP), represented by low frequency (LF) SBP, reinforces this susceptibility. The HFHSD-Sham group tended to increase LF SBP, reflecting early SNS activation induced by the diet alone. However, when these HFHSD animals underwent the additional stress of PolyhHb or Oxyglobin exchange transfusion, there was an even greater autonomic dysfunction response.

The HFHSD led to metabolic abnormalities that predisposed the animals to exaggerated SNS activation when subjected to a second hit from PolyhHb or Oxyglobin exchange transfusion. This was evidenced by greater elevation in circulating catecholamines and vascular sympathetic modulation, represented by LF SBP, in HFHSD animals receiving exchange transfusion. These findings highlight the importance of testing HBOCs such as PolyhHb or Oxyglobin in models with underlying disease conditions. The metabolic disorders caused by HFHSD increased susceptibility to cardiovascular side effects from PolyhHb or Oxyglobin exchange transfusion. Further research is warranted to determine if mitigating the diet-induced metabolic abnormalities could attenuate this exaggerated autonomic response. Testing in healthy models alone does not fully recapitulate the complexity of human disease and underlying risks for adverse events.

NO scavenging is an unavoidable problem with acellular HBOCs and, to some degree, is a side effect of PolyhHb or Oxyglobin administration. However, this effect was independent of diet. Notably, the normal diet PolyhHb group (ND-PolyhHb) showed some NO scavenging but did not exhibit increased blood pressure. This suggests that the NO scavenging itself may not be the critical factor causing vasoconstriction and hypertension in this model. We hypothesize that the increased BP in the HFHSD PolyhHb group is more likely due to autonomic nervous system impairment. Catecholamines are well known to have vasoconstrictive and hypertensive effects and appear to play a central role in elevating BP in our metabolic disorder model [27]. Although PolyhHb appears to elevate blood pressure, this effect is notably more significant in animals with diet-induced metabolic abnormalities. The blood pressure increase is relatively mild, with a 10 % rise in MAP observed in HFHSD-PolyhHb animals. While NO scavenging occurs during PolyhHb infusion, it does not entirely explain the cardiovascular effects observed. Instead, an autonomic imbalance characterized by elevated catecholamines seems to be responsible for the exaggerated hypertensive response in metabolically compromised animals receiving PolyhHb. Further research is required to determine if mitigating the catecholamine surge could alleviate the hypertension. These findings underscore the importance of evaluating HBOCs like PolyhHb in both diseased and healthy models to fully assess their safety and efficacy.

Our study yielded unexpected results in the groups that underwent transfusion with Oxyglobin. Contrary to a prior study involving this HBOC, we did not observe any significant changes in blood pressure. This discrepancy could be attributed to the isovolumic exchange we conducted, which might explain why we did not detect any alterations in BP. It’s worth noting that Rice et al. previously discussed the non-linear and possibly transient nature of blood pressure changes following Oxyglobin infusion. Given that our study primarily assessed blood pressure levels 24 hours post-infusion, it is possible that these transient fluctuations were not captured within our observations [24]. Additionally, it is important to highlight that the high blood pressure observed in animals undergoing PolyhHb exchange transfusion could be linked to the increased solution viscosity of PolyhHb. This higher viscosity may raise vascular resistance, yet conversely, it might also enhance mechanotransduction within the endothelium, stimulating the production of NO which leads to vasodilation and subsequently lowers blood pressure. In our microvascular studies utilizing the hamster window chamber model, we observed no signs of vasoconstriction with PolyhHb, contrasting with the effects noted with Oxyglobin [28,29].

Our results highlight significant implications for animals with metabolic disorders. Animals in the HFHSD group exhibited a reduced capacity to increase ferritin metabolism compared to those on a normal diet, affecting their ability to recycle excess iron following PolyhHb or Oxyglobin infusion. This limitation is likely due to endothelial dysfunction and metabolic impairments induced by HFHSD. Consequently, the HFHSD group required an enhanced breakdown of Hb into bilirubin to excrete the excess iron, as it could not be adequately recycled in this model. In contrast, ND animals effectively upregulated ferritin metabolism and did not require an increase in bilirubin levels to excrete Hb-derived iron. A previous study involving RBC transfusions in guinea pigs (GPs) with metabolic disorders and endothelial dysfunction reported increased mortality, which the authors attributed to enhanced oxidative stress [30]. They noted that iron and Hb act as potential toxic mediators in stored blood transfusions. Similarly, in our study, HFHSD animals were unable to sufficiently increase ferritin following PolyhHb infusion, likely due to their underlying endothelial and metabolic abnormalities. This has direct implications for transfusion therapy, as the excess iron from the transfused PolyhHb may leak through the dysfunctional endothelium, leading to iron accumulation and greater tissue toxicity compared to normal diet animals. The HFHSD compromises ferritin-mediated iron recycling and reuse after PolyhHb transfusion, a pattern also observed in animals exchanged and transfused with Oxyglobin. The resultant iron overload, combined with endothelial leakage, may promote oxidative damage, and exacerbate the toxicity of PolyhHb exchange transfusion in this metabolically compromised model. Further research is essential to explore strategies for mitigating these effects in disease models.

Classical markers of inflammation, liver injury, and kidney injury were evaluated. Both PolyhHb and Oxyglobin clearly affected inflammation, systemic and hepatic, independent of diet. However, organ damage was exacerbated by the presence of diet-induced metabolic alterations. This suggests the HFHSD rendered the animals less tolerant to the side effects of exchange transfusion with either PolyhHb or Oxyglobin. While exchange-transfusion induced inflammation in both diet groups, the pre-existing metabolic abnormalities caused by HFHSD appear to have compounded organ toxicity. The HFHSD GPs were overall less resilient to the inflammatory and tissue-damaging effects post-transfusion. The inflammation observed may represent a transient response to PolyhHb or Oxyglobin transfusion. Further longitudinal studies are warranted to evaluate if inflammatory markers can return to normal levels over an extended period post-transfusion. PolyhHb and Oxyglobin induce inflammation in both healthy and metabolically compromised animals, but pre-existing metabolic dysfunction exacerbates subsequent organ damage. Testing HBOCs such as PolyhHb in disease models is important to fully characterize toxicity risks and identify opportunities to improve safety through mitigation of diet-induced abnormalities. Follow-up studies on the resolution of inflammation over time could further inform the transient nature of these effects.

Evaluation of cardiac markers showed a similar pattern. PolyhHb induced inflammation to a comparable degree in both diet groups. However, HFHSD animals exhibited greater elevation in cardiac troponin, an important marker of cardiac injury, after PolyhHb infusion. A similar effect was observed with ANP, though statistical significance was not reached. Importantly, Oxyglobin appeared more cardiotoxic than PolyhHb in both ND and HFHSD groups. This highlights the improved safety profile of the new generation PolyhHb compared to prior HBOC formulations. While PolyhHb induced inflammatory effects across groups, pre-existing metabolic dysfunction in HFHSD animals led to exacerbated cardiac tissue injury compared to healthy ND controls when challenged with a PolyhHb infusion. The HFHSD appears to increase susceptibility to cardiac damage from PolyhHb, though PolyhHb still showed reduced cardiotoxicity compared to Oxyglobin in both diet groups. This highlights the importance of testing in models of disease to fully evaluate the safety of HBOCs.

Although the HFHSD group experienced greater physiological alterations when subjected to PolyhHb exchange transfusion, these results provide important insights into the effects of PolyhHb in a model reflecting a large segment of the population with pre-existing metabolic disease. The diet-induced pre-existing abnormalities led to exaggerated responses to PolyhHb transfusion across several parameters. However, PolyhHb was still better tolerated than the older generation Oxyglobin in this metabolically compromised model. These findings can inform future strategies to mitigate the side effects of PolyhHb transfusion in populations with underlying diseases. For example, ascorbic acid supplementation could be explored for protective effects against PolyhHb-mediated oxidative stress and inflammation. Although this study revealed increased physiological disturbances in unhealthy animals, PolyhHb still showed advantages over prior HBOCs. These data can guide continued optimization and risk reduction of PolyhHb as a novel RBC substitute. Evaluating both benefits and risks in disease models is key for successful clinical development.

## 5. Conclusion

In conclusion, this study demonstrates that metabolic disorders exacerbated certain side effects of PolyhHb or Oxyglobin exchange transfusion. However, high MW PolyhHb showed less cardiac toxicity than prior generations of HBOCs, such as Oxyglobin, even in animals with pre-existing metabolic abnormalities. These findings highlight the importance of testing blood substitutes such as high MW PolyhHb in disease models to fully characterize safety and optimize efficacy. This study provides key insights that can guide the continued improvement of high MW PolyhHb as an emergent blood replacement therapy for broad use in complex patient populations.

## Supplementary Material

supplemental

## Figures and Tables

**Table 1:** HBOC biophysical properties.

Solution	Protein Concentration (g/dL)	MetHb Level (%)	Average Molecular Weight (kDa)	P50 (mm Hg)
**PolyhHb**	10.6	5.8	> 500 kDa < 0.2 μm	41
**Oxyglobin**	12.2	1	Up to 500 kDa	42

**Table 2:** Hematological parameters.

	ND-Sham	ND-PolyhHb	ND-Oxyglobin	HFHSD-Sham	HFHSD-PolyhHb	HFHSD-Oxyglobin
**tHb (g/dL)**	14.9±0.2	12.1 ± 0.3^†^	12.2 ±0.5^†^	14.6±0.2	11.6±0.3^†^	12.0±0.3^†^
**pH**	7.449±0.017	7.471±0.008	7.461±0.009	7.468±0.012	7.468±0.008	7.445±0.014
**pCO**_**2**_ **(mmHg)**	38.9±1.5	36.1±1.1	36.8±1.4	34.6±0.9*	32.8±1.1*	33.2±1.2
**pO**_**2**_ **(mmHg)**	60.4±6.0	67.5±4.2	69.1±4.8	71.9±3.8	73.7±3.8	74.4±4.8
**K**^**+**^ **(mmol/L)**	3.8±0.1	4.0±0.1	3.9±0.1^†^	3.7±0.1	3.8±0.1	3.9±0.1
**Na**^**+**^ **(mmol/L)**	145.6±0.3	144.5±0.8	144.1±1.3	139.8±1.0*	139.9±1.1*	140.9±1.1*
**Ca**^**+**^ **(mmol/L)**	1.15±0.02	1.18±0.04	1.1±0.04	1.12±0.05	1.06±0.02	1.1±0.05
**Cl^−^ (mmol/L)**	112.1±0.7	113.2±0.8	112.3±1.7	106.7±1.2*	108.2±1.2*	110.8±0.8^†^
**Lactate (mmol/L)**	0.64±0.07	0.66±0.04	0.68±0.06	0.57±0.04	0.68±0.04	0.95±0.14*^†^‡

Data presented as mean ± SE. Normal diet (ND), and high-fat high-sucrose diet (HFHSD). Sham or 20% exchange transfused with PolyhHb or Oxyglobin.

* p< 0.05 compared ND.

† < 0.05 compared to Sham.

‡ p< 0.05 compared to PolyhHb.

ND-Sham (n =8); ND-PolyhHb (n=12); ND-Oxyglobin (n=9); HFHSD-Sham (n = 9); HFHSD-PolyhHb (n=10); and HFHSD-Oxyglobin (n=12).

**Table 3:** Inflammation, catecholamines, and iron metabolism.

	ND-Sham	ND-PolyhHb	ND-Oxyglobin	HFHSD-Sham	HFHSD-PolyhHb	HFHSD-Oxyglobin
**IL-6 (pg/mL)**	185.9±4.0	287.7±8.6^†^	287.9±6.6^†^	185.8±12.8	355.2±14.1*^†^	351.1±11.5*^†^
**IL-10 (pg/mL)**	186.4±6.4	305.4±22.1^†^	288.6±12.0^†^	202.3 ± 6.7	360.2±18.4^†^	344.1±9.7*^†^
**CXCL-1 (pg/mL)**	119.0±4.4	170.9±11.6^†^	191.8±7.0^†^‡	124.7±1.4	233.6±7.1*^†^	232.7±6.4*^†^
**Spleen CXCL1 (pg/mg)**	210.2±9.3	354.3±11.7^†^	354.2±11.4^†^	246.6±13.3	440.6±11.4*^†^	456.1±18.0*^†^
**Norepinephrine (pg/mL)**	699.0±16.2	775.2±34.7	846.1±27.5^†^	732.4±16.4	1039.5±48.3*^†^	1093±29.9*^†^
**Epinephrine (pg/mL)**	224.4±17.6	293.9±9.9^†^	331.3±8.4^†^	271.6±11.2*	414.5±21.4*^†^	398.8±10.2*^†^
**Serum ferritin (μg/L)**	235.9±10.6	396.3±25.6^†^	400.7±10.3^†^	228.3±5.8	287.2±2.4*^†^	289.3±8.8*^†^
**Liver ferritin (μg/g)**	210.4±15.1	483.5±40.4^†^	491.2±15.2^†^	224.8 ± 8.3	299±20.1*^†^	292.7±11.4*^†^
**Spleen ferritin (μg/g)**	305.3±26.9	712.2±70.5^†^	748.5±19.8^†^	278.8±11.5	408.7±23.7*^†^	397.2±10.2*^†^
**Total bilirubin (mg/dL)**	5.0±0.3	5.8±0.4	6.3 ± 0.1^†^	6.1±0.2*	10.1±0.1*^†^	10.5±0.3*^†^

Data presented as mean ± SE. Normal diet (ND), and high-fat high-sucrose diet (HFHSD) for the Sham group or 20 % exchange transfusion wih the PolyhHb or Oxyglobin groups.

* p< 0.05 compared to ND.

† < 0.05 compared to Sham.

‡ p< 0.05 compared to PolyhHb.

ND-Sham (n =8); ND-PolyhHb (n=12); ND-Oxyglobin (n=8); HFHSD-Sham (n = 9); HFHSD-PolyhHb (n=10); HFHSD-Oxyglobin (n=8).

## Appendix A. Supporting information

Supplementary data associated with this article can be found in the online version at doi:10.1016/j.biopha.2024.116789.
