# Supplementary material for: Novel high molecular weight polymerized hemoglobin in a non-obese model of cardiovascular and metabolic dysfunction
Source: Biomed Pharmacother. Author manuscript; Available in PMC 2026 Feb 9. (PMC12885157; doi:10.1016/j.biopha.2024.116789)
Supplement: supplemental [file NIHMS2138936-supplement-supplemental.pdf]

Supplementary Material

Novel high molecular weight polymerized hemoglobin in a non-obese model of cardiovascular and metabolic dysfunction.

Supplementary Table 1. ELISA kits used in the analysis.

Method	Kit/Assay	Analyte	Vendor
ELISA	KA1625	AST	Abnova Corp, Taiwan
ELISA	KA4189	ALT	Abnova Corp, Taiwan
ELISA	BMS625	IL-6	Thermo Fisher, Waltham, MA
ELISA	BMS629	IL-10	Thermo Fisher, Waltham, MA
ELISA	KB02-H2	Creatinine	Arbor Assays Inc, Ann Arbor, MI
ELISA	K024-H5	BUN	Arbor Assays Inc, Ann Arbor, MI
ELISA	ERCXCL1	CXCL1	Thermo Fisher, Waltham, MA
ELISA	MCA-155	Ferritin	Serotec, Oxford, UK
ELISA	BA-E-6600	Catecholamines	ImmunoSmol, France
ELISA	ERLCN2	Urine NGAL	Thermo Fisher, Waltham, MA
ELISA	ab235627	Bilirubin	Abcam, Cambridge, UK
Luminex technology /Miliplex Immunoassay	RECYTNMAG-65K	MCP-1	Millipore Corporation, Massachusetts, USA
Luminex technology /Miliplex Immunoassay	RECYTNMAG-65K	TNF-α	Millipore Corporation, Massachusetts, USA
ELISA	ab246529	Cardiac Troponin	Abcam, Cambridge, UK
ELISA	ab256398	CRP	Abcam, Cambridge, UK
ELISA	ab108797	ANP	Abcam, Cambridge, UK

Supplementary Table 2. Heart rate, blood pressure variability and spectral analysis.

	ND-Sham	ND-PolyhHb	ND-Oxyglobin	HFHSD-Sham	HFHSD-PolyhHb	HFHSD-Oxyglobin
**HRV SD (ms)**	11.1±1.2	12.65±1.87	7.97±1.15	12.9±1.6	9.98±1.50	14.21±1.94*
**HRV Variance (ms^2)**	120.4±21.3	195.0±54.1	71.4±19.8	196.7±49.4	115.5±31.0	236.0±70.0*
**RMSSD (ms)**	9.0±1.0	7.4±1.0	6.0±1.0	11.2±1.3	7.4±1.0†	10.0±1.1*
**LF abs (ms^2)**	27.0±6.1	31.0±7.8	30.9±6.8	37.7±9.4	28.6±10.2	14.9±2.5†
**HF abs (ms^2)**	27.2±5.6	12.6±2.7†	20.1±6.0	33.0±6.8	11.8±2.4†	29.0±2.5
**LF %**	26.6±3.3	39±4.5†	23.9±3.9‡	32.4±3.7	37.9±4.2	32. 0±3.4
**HF %**	42.1±4.1	28.5±4.7†	29.9±5.8	41.3±5.5	31.0±3.9	31.0±3.3
**LF (nu)**	43.4±4.2	59.9±5.7†	63.4±6.0 †	46.4±5.35	55.4±5.1	33.3±3.9 *‡
**HF (nu)**	59.1±4.6	40.1±5.7†	36.6±6.0†	53.6±5.4	44.5±5.1	66.7±3.9 *‡
**LF/HF**	1.2±0.2	2.7±0.6†	2.3±0.7†	1.4±0.3	1.7±0.3	0.6±0.1 *‡
**BPV SD (mmHg)**	4.4±0.2	5.0±0.4	5.0±0.3	5.7±0.5 *	6.0±0.3	6.5±0.5*
**BPV Variance ($mmHg^2$)**	19.9±2.0	26.2±3.8	25±3.4	35.6±5.8*	36.4±3.6	43.8±6.0*

Data presented as mean ± SE. Normal diet (ND), and high-fat high-sucrose diet (HFHSD) for the Sham group or 20% exchange transfusion with PolyhHb or Oxyglobin groups. *p< 0.05 compared to ND, † < 0.05 compared to Sham. ‡ p< 0.05 compared to PolyhHb. ND-Sham (n =17); ND-PolyhHb (n=11); ND-Oxyglobin (n=7); HFHSD-Sham (n = 13); HFHSD-PolyhHb (n=8); HFHSD-Oxyglobin (n=9).
